# Glycosylation of Zein Hydrolysate as a Nanocarrier for Lutein Delivery: Preparation and Stability

**DOI:** 10.3389/fphar.2022.905059

**Published:** 2022-05-02

**Authors:** He Han, Yan Jiao, Ying Chang, Yue Cheng, Lei Shi

**Affiliations:** College of Food and Bioengineering, Qiqihar University, Qiqihar, China

**Keywords:** glycosylated zein, hydrolysate, lutein nanocarrier, preparation, stability

## Abstract

Lutein is a functional carotenoid that has a wide range of physiological benefits in humans. However, it easily degrades and becomes inactivated during storage and processing, resulting in low bioavailability. The development of new nanocarriers can effectively improve the stability and biological activity of lutein. In this study, zein hydrolysate (ZH) carriers were glycosylated with glucosamine (GLU) under the action of transglutaminase, and lutein-loaded glycosylated ZH nanoparticles (GZH-LUT) were constructed by liquid–liquid dispersion. The results showed that the GZH-LUT particles had a narrow size distribution in the range of 200–300 nm and a decreased zeta potential and polydispersity index. In particular, GZH trapped lutein more efficiently than ZH. In addition, GZH-LUT had better physical and chemical properties, including better water solubility, oxidative stability, and environmental stability than free lutein and ZH-LUT. These results indicate that glycosylated zein hydrolysate has the potential to be used as a novel protein-based nanocarrier to enhance the solubility and stability of lutein, which can further improve its bioavailability.

## 1 Introduction

Because the concept of a “healthy life” is deeply rooted in the hearts of the people, people’s demand for food nutrition and health is increasing (Belyaeva et al., 2021), and many biologically active substances are widely used in various food systems. They can significantly improve human immunity and play a role in preventing diseases. However, most bioactive substances have high hydrophobicity and low bioavailability and are easily oxidized and degraded by light and heat during production and storage. The use of nanotechnology to encapsulate biologically active substances can effectively protect their integrity and stability (Milinčić et al., 2019; Ijaz et al., 2020), prevent degradation during processing or storage, and improve their efficiency in the human body (Lisitskaya et al., 2012; Wang P. et al., 2020; Naseer et al., 2018). Therefore, nanotreatment is performed on biologically active substances, which has very important practical significance for promoting body absorption and further exerting its biological activity (McNamara et al., 2017; Melo van Lent et al., 2016).

Lutein (3.3-dihydroxy-α-carotene) is a natural hydrophobic pigment in the lutein family, and it is also the main pigment in the human macula (Su et al., 2020; Santos et al., 2021). The molecular formula of lutein is C_40_H_50_O_2_, and the molecular weight is 568.85. Lutein is a terpenoid that widely exists in vegetables (Fu et al., 2019), flowers, fruits and other plants and has the physiological functions of antioxidation, antiradiation, cancer prevention and other physiological functions (Stringham et al., 2019; Widomska et al., 2020). In addition, lutein can also effectively reduce some age-related diseases, such as macular degeneration and senile cataracts (Melo van Lent et al., 2016; Ramirez, 2016). Animals and humans cannot synthesize lutein by themselves, so it must be obtained from external diets (Granado et al., 2003).

Unfortunately, due to the multiple unsaturated double bonds in the main chain structure, lutein is highly unsaturated (Ma et al., 2021), has strong lipid solubility and is chemically unstable, which leads to low bioavailability (Shegokar and Mitri, 2012; Elvira-Torales et al., 2019; Demmig-Adams et al., 2020). To fully enable the antioxidant, antiradiation and anticancer properties of lutein (Chittasupho et al., 2018), it is necessary to develop a nanocarrier system for lutein encapsulation to increase the physicochemical stability of lutein under processing and storage.

Zein is a safe, nontoxic, renewable, natural, low-cost, biocompatible and degradable protein. It is the main storage protein in corn, accounting for 45–50% of corn protein (Yavari et al., 2021). It is composed of four components (α, β, γ and δ), which have different peptide chains, molecular sizes and solubilities. In practice, zein hydrolysate, which contains hydrophobic and hydrophilic groups (Ma et al., 2020), is amphiphilic, and the nonpolar amino acids of zein hydrolysate themselves can be self-assembled into spherical particles to be an ideal delivery matrix for bioactive compounds (Kasaai et al., 2018; Li H. et al., 2020), drugs, oils and other nutritional foods and food components (Elisângela et al., 2014). However, colloidal zein particles are large, and they easily aggregate in the aqueous phase and have poor controlled release performance for loaded fat-soluble functional ingredients (Dai et al., 2017; Huang et al., 2019), which greatly limits the application of zein in the field of functional carriers (Wang Y.-H. et al., 2017). At present, some studies have found that the structure of zein modified by enzymatic hydrolysis can improve the functional characteristics of the protein and make it more suitable as an effective carrier of functional active substances (Wang et al., 2015; Jiao et al., 2018; Xu Y. et al., 2020; Liu et al., 2021), thus improving the loading performance of fat-soluble lutein. However, the nano transport system of a single protease hydrolysate still has many shortcomings in practical applications, such as poor thermal stability and sensitivity to environmental pH changes.

The glycosylation modification of the protein structure and subsequent improvement of some functional properties of the initial protein is of recent interest (Gaber et al., 2018). The principle of glycosylated proteins is to graft small sugars to protein macromolecules through covalent bonding so that the glycosylated products have both the functional properties of the initial protein and the hydrophilic properties of sugars (Bollmann et al., 2011; Chen and Kitts, 2015). In general, there are two main ways to modify protein glycosylation (Liu and Zhong, 2012; Hafsa et al., 2021): the Maillard reaction and the transglutaminase enzyme reaction, glycosylation. The Maillard reaction is a nonenzymatic browning reaction between amino compounds and carboxyl compounds (Ferruzzi et al., 2012), also known as the carboxyl-amino reaction. The Maillard reaction can significantly increase the initial colour, emulsification, soluble protein, thermal stability, and antioxidant activity. Modification of the protein carrier by the Maillard reaction can enhance the stability of the protein and nanodelivery system of the protein carrier under certain environmental conditions (Chen and Kitts, 2012; Ferruzzi et al., 2012). However, the Maillard reaction may produce carcinogenic and mutational substances in the modification process, which can harm human health (Su et al., 2020; Kaczynska et al., 2022). In contrast, enzymatic glycosylation reaction conditions are relatively mild, and the product is relatively safe and has the advantages of a high rate and strong specificity. Therefore, this method has attracted much attention. In addition, due to the intervention of sugar molecules, the initial protein polypeptide carrier on the surface and internal part of the hydrophobic microregion is blocked, the whole protein polypeptide carrier internal hydrophobic core increases, the central active material embedding capacity gradually improves, and the modified protein polypeptide carrier thermal stability, antioxidant activity and bioavailability and other functional characteristics significantly improve (Liu and Zhong, 2012; Lin et al., 2018).

In this study, zein hydrolysate was modified by glycosylation with glucosamine to develop a new nanocarrier. Lutein-loaded ZH and GZH nanoparticles (ZH-LUT and GZH-LUT) were prepared, and the structural characteristics, water solubility, oxidative stability, and environmental stability of the nanoparticles were also evaluated.

## 2 Materials and Methods

### 2.1 Materials

Lutein (≥90%), glucosamine, and zein were obtained from Sigma Company (St. Louis, MO, United States). Neutral protease (50,000 U/g) and transglutaminase (TGase) (1,000 U/g) were purchased from Shanghai Blue Season Biological Co., Ltd. Anhydrous ethanol and petroleum ether were analytically pure. All other chemicals used were of analytical grade and used without further purification. Ultrapure water was used in all experiments.

### 2.2 Preparation of Zein Hydrolysate

The zein was hydrolysed by neutral protease (NPT). A total of 1.0 g of zein was mixed with deionized water, 2.7% neutral protease was added to the solution (W/W, substrate weight calculated by protein mass), the pH of the solution was adjusted to 6.0, and hydrolysis was carried out at 50°C for 60 min. After hydrolysis, the reaction was terminated by boiling the mixture at 100°C for 10 min. The zein hydrolysate was freeze-dried for glycosylation use (Kongo-Dia-Moukala et al., 2011; Ma et al., 2012; Jin et al., 2016).

### 2.3 Preparation of Glycosylated Zein Hydrolysate

To prepare an aqueous solution containing 5% ZH and 15% glucosamine, the pH of the reaction system was adjusted to 7.5 with 1 mol/L HCl solution and 2 mol/L NaOH solution, and 0.15% TGase was added. The glycation reaction system was placed in a constant temperature oscillation incubator, the reaction was conducted at 40°C for 7 h, and the pH was adjusted every 30 min. After the reaction was complete, the enzyme was inactivated at 95°C for 5 min, and the reaction solution was then dialyzed at 4°C for 48 h to remove the unreacted glucosamine. The samples were freeze-dried as glycosylated zein hydrolysate (GZH), which was stored at 4°C for later use (Wang et al., 2016; Wang XJ. et al., 2017; Kaczynska et al., 2022).

### 2.4 Preparation of ZH-LUT and GZH-LUT Nanoparticles

**FIGURE 1:**
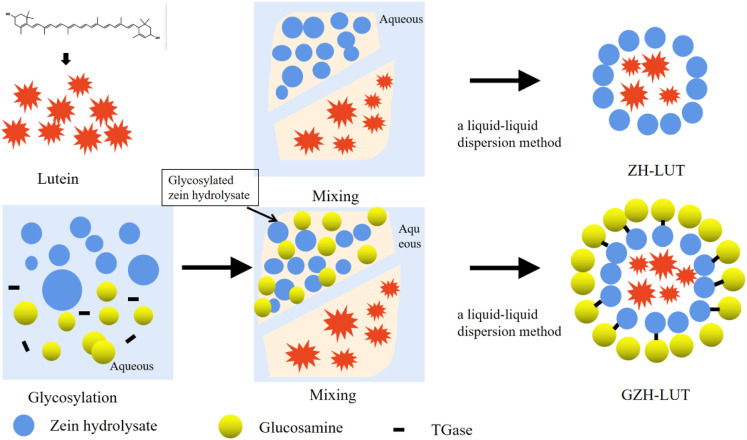
Schematic diagram for the preparation of ZH-LUT and GZH-LUT.

The GZH-LUT nanoparticles were prepared by liquid–liquid dispersion. First, 5.0 ml of a standard solution of lutein (100 μg/ml) was added to 5.0 ml of an ethanol solution of GZH (2.5 mg/ml), and the mixture was thoroughly mixed. Centrifugation was performed at 3,000 r/min for 5 min. The supernatant was added to 18.5 ml of PBS buffer solution and stirred at 45°C for 30 min. The GZH-LUT system was transferred to a rotary evaporator, and ethanol was removed under reduced pressure to obtain a GZH-LUT nanoparticle solution (Li S. et al., 2020; Zhang R. et al., 2022; Liu Q. et al., 2022). The ZH-LUT nanoparticles were prepared using the same procedure. The free lutein suspension made with the aqueous phase followed the same protocol, which served as a control (The preparation schematic diagram was shown in Figure 1).

### 2.5 Entrapment Efficiency

Aliquots of 3.0 ml of ZH-LUT or GZH-LUT and 3 ml of petroleum ether were mixed by vortexing them vigorously for 5 min at ambient temperature. The mixed samples were then centrifuged at 4,000 r/min for 5 min to collect the supernatant of the centrifuged sample. The above operation was repeated three times. Finally, the collected supernatant was transferred to a test tube, and the amount of free lutein was determined by UV/vis spectrophotometry (Shanghai Jinghua Technology Instrument Co., Ltd., China) at 445 nm. The lutein concentration was calculated according to the standard curve of lutein (Y = 0.005X+0.0005, R^2^ = 0.9991) (De Boer et al., 2020; Boostani et al., 2022). All measurements were performed in triplicate.

$$\text{EE}(\%)=\left(1-\frac{\text{the weight of the free amount of lutein}}{\text{The initial weight of lutein added into the preparation}}\right)\times 100\text{\%}$$ (1)

### 2.6 Morphology Analysis

The shapes and characteristics of the ZH-LUT and GZH-LUT nanoparticles were visualized by TEM (Hitachi H-7650, Tokyo, Japan) (Hanna et al., 2020). The freshly prepared dispersions were diluted with water, and one drop of the diluted dispersion was placed on a 200-mesh carbon-coated copper grid. Images of the nanoparticles were captured at 100 kV with a magnification of 50,000 (Ouyang et al., 2020).

### 2.7 Particle Size, Polydispersity Index, and Zeta Potential Analyses

Fresh ZH-LUT and GZH-LUT nanoparticles were characterized by measuring the average diameter size, PDI, and zeta potential by dynamic light scattering (DLS) using a Zetasizer Nano ZS90 (Malvern Instruments Ltd., Worcestershire, United Kingdom) Nanocomplex samples were prepared by diluting the stock samples 1:20 with phosphate buffer (10 mM, pH 7.0), and filtered through a 0.45 μm filter. The purpose of dilution was to minimize the multiple scattering effects of the instrument (Bhawana et al., 2011; Wang Q. et al., 2017). All measurements were performed in triplicate.

### 2.8 Fourier Transform Infrared Spectroscopy

The Fourier transform infrared spectra (FTIR) were recorded by IR spectroscopy (PE, America). Nanocomplex samples were blended with KBr at a mass ratio of 1:100 and pressed into a tablet for FTIR analysis. The infrared spectra of pure potassium bromide were set as the background of all samples. The plate was scanned by the infrared spectrometer from 4,000 to 400 cm^−1^ (Tian et al., 2018). The infrared spectra of lutein, ZH-LUT, and GZH-LUT nanoparticles were recorded and analysed.

### 2.9 Solubility

The effects of ZH and GZH carriers on the solubility and dispersibility of lutein were investigated. To determine the solubility of lutein, the lutein, ZH-LUT and GZH-LUT nanoparticles were centrifuged at 5,000 r/min for 10 min, and the supernatant was filtered through a 0.45 m membrane to remove the insoluble lutein. The filtered solution (V_sample_) was ultrasonically shaken for 5 min, 2.0 ml of sample was mixed with 4.0 ml of petroleum ether (V_1_), and the mixture was vortexed for 60 s. The experiment was repeated three times. The concentration of lutein solution C_2_ was calculated. The solubility is calculated according to the formula (Chang et al., 2017; Chen et al., 2020).

$$\text{Solubility}=\frac{{\text{C}}_{2}\times {\text{V}}_{1}}{{\text{V}}_{\text{sample}}\;}$$ (2)

### 2.10 Stability to Environmental Stresses

#### 2.10.1 Heat Stability

The lutein, ZH-LUT and GZH-LUT nanoparticles were incubated in a water bath set at different temperatures (4, 20, 37, 50, 70°C) for 2.0, 4.0, 6.0, 8.0, 10.0, and 12.0 h. A total of 1.0 ml was withdrawn from each sample and then extracted and analysed by measuring the lutein concentration according to the method of 2.5, and the mean particle size (Dz, nm) was then determined by DLC (Malvern Instruments Ltd., Worcestershire, United Kingdom) (Sun et al., 2014; Chittasupho et al., 2018). The degradation rates were calculated to compare heat stability.

#### 2.10.2 pH Stability

All samples were added to phosphate buffer solutions at a different pH (2.5, 5, 6, 7.4, 8) and reacted in the dark for 12 h. The pH stability of lutein, ZH-LUT and GZH-LUT nanoparticles was evaluated by studying these. The pH was adjusted with NaOH (0.2 M) and HCl (0.10 M) when needed (Li H. et al., 2020). The mean particle size and lutein content of the samples were monitored and compared to analyse the pH stability.

#### 2.10.3 Light Stability

All nanoparticles were placed in transparent glass vials and stored in a light radiation-proof cabinet where they were exposed to 365 nm UV lamps for up to 12 h. At exposure time intervals (2, 4, 6, 8, 10, and 12 h), the mean particle size and free lutein content of the samples were monitored to analyse the light stability of lutein in the ZH-LUT and GZH-LUT nanoparticles (Chuacharoen et al., 2016).

### 2.11 Antioxidant Capacity

#### 2.11.1 DPPH Radical Scavenging Activity

Lutein, ZH-LUT and GZH-LUT nanoparticles at different concentrations (2.0 ml) were added to 2.0 ml of 0.8 mg/ml DPPH solution, which was fully shaken and reacted in the dark for 30 min, and the absorbance was read at 517 nm. The absorbance at 517 nm was recorded as A_1_. Only ethanol was added to the sample as a blank group, recorded as A_2._ Ethanol was used as a control group instead of the sample, recorded as A_0_. The radical scavenging activity (AAD) was calculated according to the following equation (Lin et al., 2019):

$$\text{AAD}\left(\text{\%}\right)=\left[1-\frac{{\text{A}}_{1}-{\text{A}}_{2}}{{\text{A}}_{0}}\right]\times 100\%$$ (3)

#### 2.11.2 Hydroxyl Radical Scavenging Ability

Lutein, ZH-LUT and GZH-LUT nanoparticles at different concentrations (0.2 ml) were mixed with l.0 ml of 6 mmol/L FeSO_4_ and 1 ml of 6 mmol/L salicylic acid solution and mixed well. Then, 1.0 ml of 6 mmol/L H_2_O_2_ solution was added to initiate the reaction, and the mixture was incubated in the dark for 30 min. The absorbance 510 nm was recorded as A_1_, the FeSO_4_ solution was replaced with 1.0 ml of distilled water and recorded as A_2_, and the nanosuspension was replaced with 0.2 ml of distilled water and recorded as A_0_. The radical scavenging activity (AAH) was calculated according to the following equation (Wijewickreme et al., 1999):

$$\text{AAH}\left(\text{\%}\right)=\left[1-\frac{{\text{A}}_{1}-{\text{A}}_{2}}{{\text{A}}_{0}}\right]\times 100\%$$ (4)

### 2.12 Statistical Analysis

All data are presented as the mean ± standard deviation (±SD). Statistical analysis was performed using one-way ANOVA with *p* < 0.05 considered significant. Calculations were performed using SPSS 17.0 software.

## 3 Results and Discussion

### 3.1 Entrapment Efficiency

**FIGURE 2:**
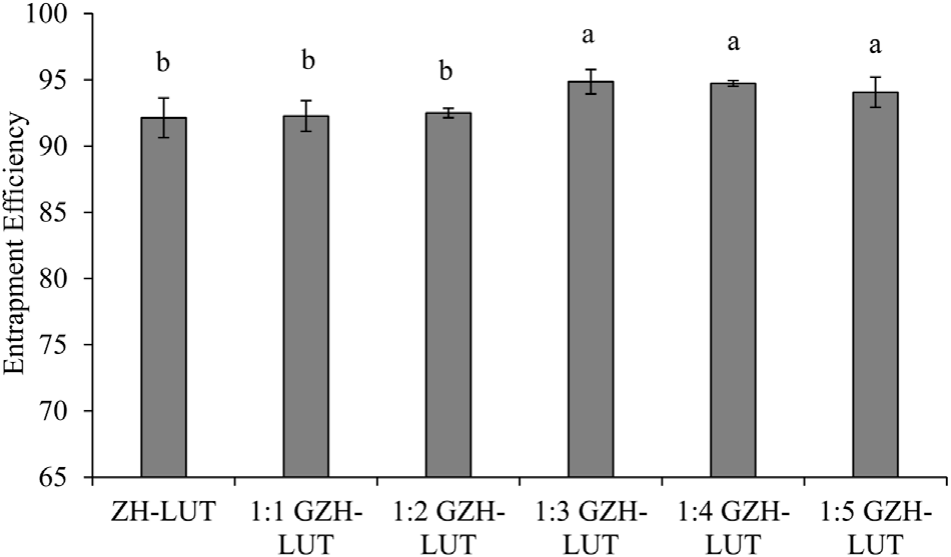
Encapsulation efficiency of ZH-LUT and GZH-LUT.

With the increase in the molecular weight of sugar, the degree of glycosylation reaction gradually intensified, hydrophobic side chains of ZH carriers were gradually exposed, hydrophobic kernels in the inner core of the ZH carriers were gradually formed, the internal molecular structure of the ZH carriers was partially expanded, and the capture ability of lutein was gradually enhanced. Glucosamine was grafted onto ZH carriers, which made the ZH carriers undergo stronger hydrophobic interactions and hydrogen bonding, which was beneficial to the combination of the ZH carriers and lutein, thereby improving the encapsulation ability of the ZH carriers (Liu et al., 2020a; Liu J. et al., 2022). The encapsulation efficiency of GZH-LUT nanoparticles reached the maximum when the mass ratio of ZH carriers and glucosamine was 1:3 (Figure 2), and the structure of ZH carriers changed little when the number of sugar molecules was increased. However, increasing the number of sugar molecules would also increase the production cost.

### 3.2 Transmission Electron Microscopy

**FIGURE 3:**
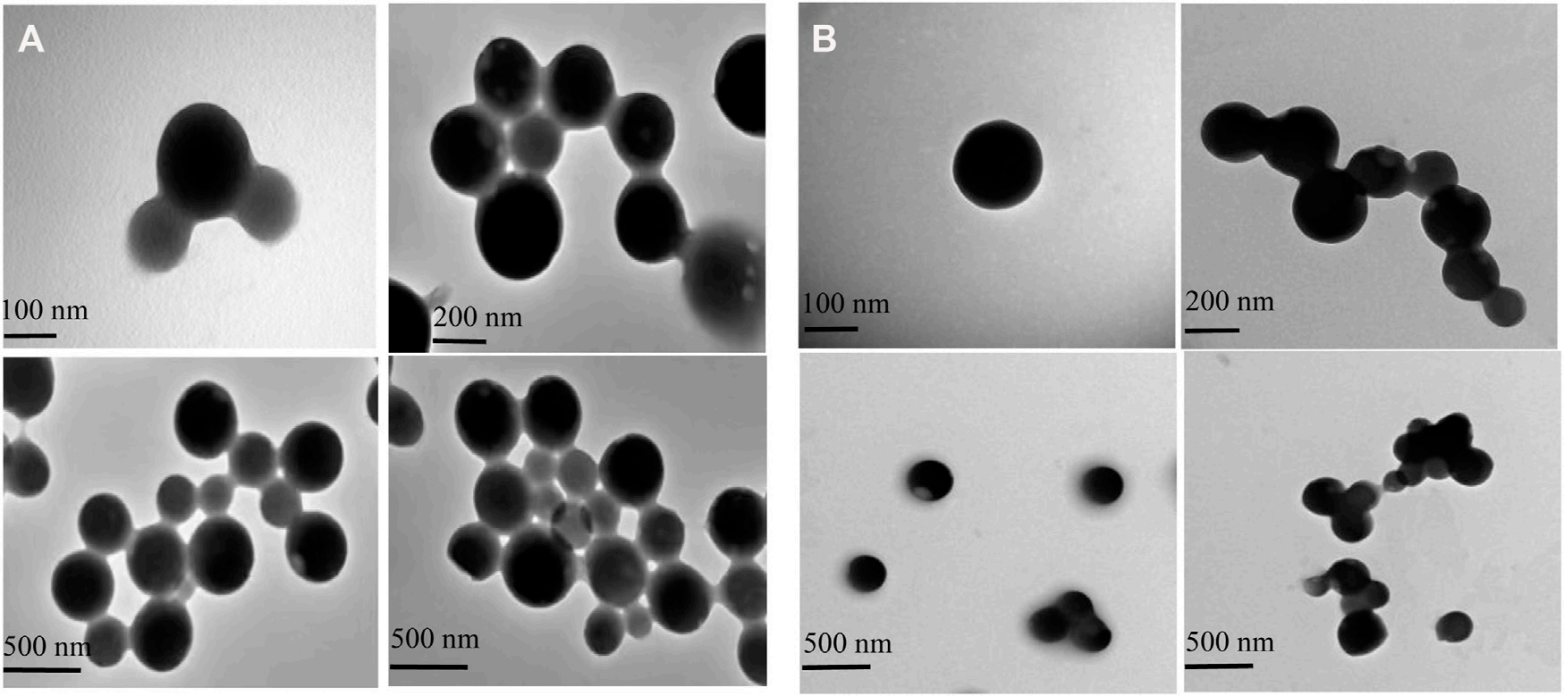
Transmission electron microscopy (TEM) images of ZH-LUT **(A)** and GZH-LUT **(B)**. The freshly prepared ZH-LUT, GZH-LUT nanoparticles were diluted 20 times with water, and a drop of the diluted nanoparticles was placed on a 200 mesh carbon-coated copper grid. Images of the nanoparticles were taken at 100 kV at a certain magnification. The scale bar represents 100, 200, and 500 nm in both **(A)** and **(B)**.

The structure of the GZH-LUT nanoparticles was similar to that of the ZH-LUT nanoparticles, with a spherical structure in appearance. After glycosylation, the surface of the GZH carriers has a large number of negative charges, and the repulsive force between them increased, therefore, the dispersion of GZH-LUT nanoparticles was relatively good (Chuacharoen et al., 2016; Wang H. et al., 2020). After grafting the ZH carriers with glycosylation, the molecular weight increased, and therefore the size of the GZH-LUT nanoparticles was larger than that of the ZH-LUT nanoparticles, which was consistent with the results of the nanoparticle size analyzer (Figure 3).

### 3.3 Particle Size, Polydispersity Index, and Zeta Potential Analyses

**FIGURE 4:**
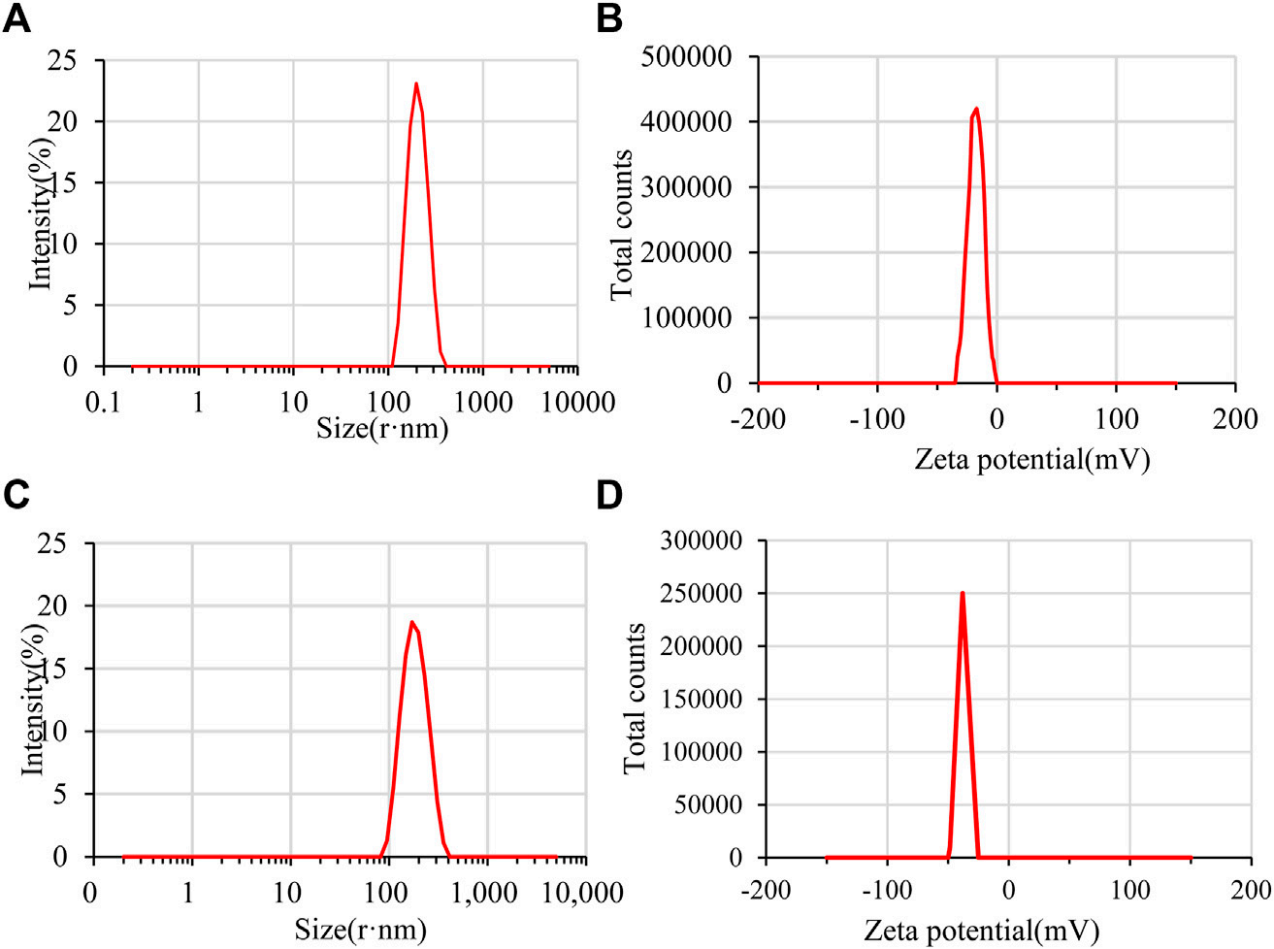
Particle size and zeta potential of ZH-LUT **(A,B)** and GZH-LUT **(C,D)**.

The particle size range of GZH-LUT nanoparticles was 127.5–477.7 nm, and the average particle size was 218.4 ± 8.41 nm, which was larger than that of ZH-LUT nanoparticles (173.7 ± 4.75 nm) (Figures 4A,C). The reason may be that the molecular weight of GZH-LUT nanoparticles increased after glycosylation and grafting, meaning that they were larger in size (Meng et al., 2021; Zhang T. et al., 2022). 2. The encapsulation efficiency of the GZH-LUT nanoparticles increased, which increased the hydrophobic core of the GZH carriers, thereby increasing the average particle size of the nanoparticles.

PDI is usually used to reflect the particle size distribution of the particles (Brum et al., 2017). When the PDI of the prepared nanoparticles is less than 0.3, it indicates that the nanosystem is well dispersed. The PDI of ZH-LUT nanoparticles and GZH-LUT nanoparticles were 0.079 ± 0.018 and 0.077 ± 0.033, respectively. This result indicates that the two kinds of nanoparticles formed a good dispersion system.

The zeta potential can indicate the potential stability of a colloidal system (Bolla et al., 2020). Generally, the smaller the absolute value of the zeta potential of the nanoparticles is, the easier the cohesion occurs between the nanoparticles, and as the absolute value of the potential increases, the repulsive force between particles increases; when the particles do not flocculate, the nanosystem is more stable (Li S. et al., 2020). The zeta potential of the GZH-LUT nanoparticles was -32.1 ± 4.06 mV, which was much lower than that of the ZH-LUT nanoparticles (-18.1 ± 4.2 mV) (Figures 4B,D). Because the surface of the modified ZH carrier had a large number of negative charges, the repulsive force between the charges increased. Therefore, the zeta potential of GZH-LUT nanoparticles increased. This change is helpful for improving the stability of the nanoparticles (Toragall et al., 2020).

### 3.4 Fourier Transform Infrared Spectroscopy

**FIGURE 5:**
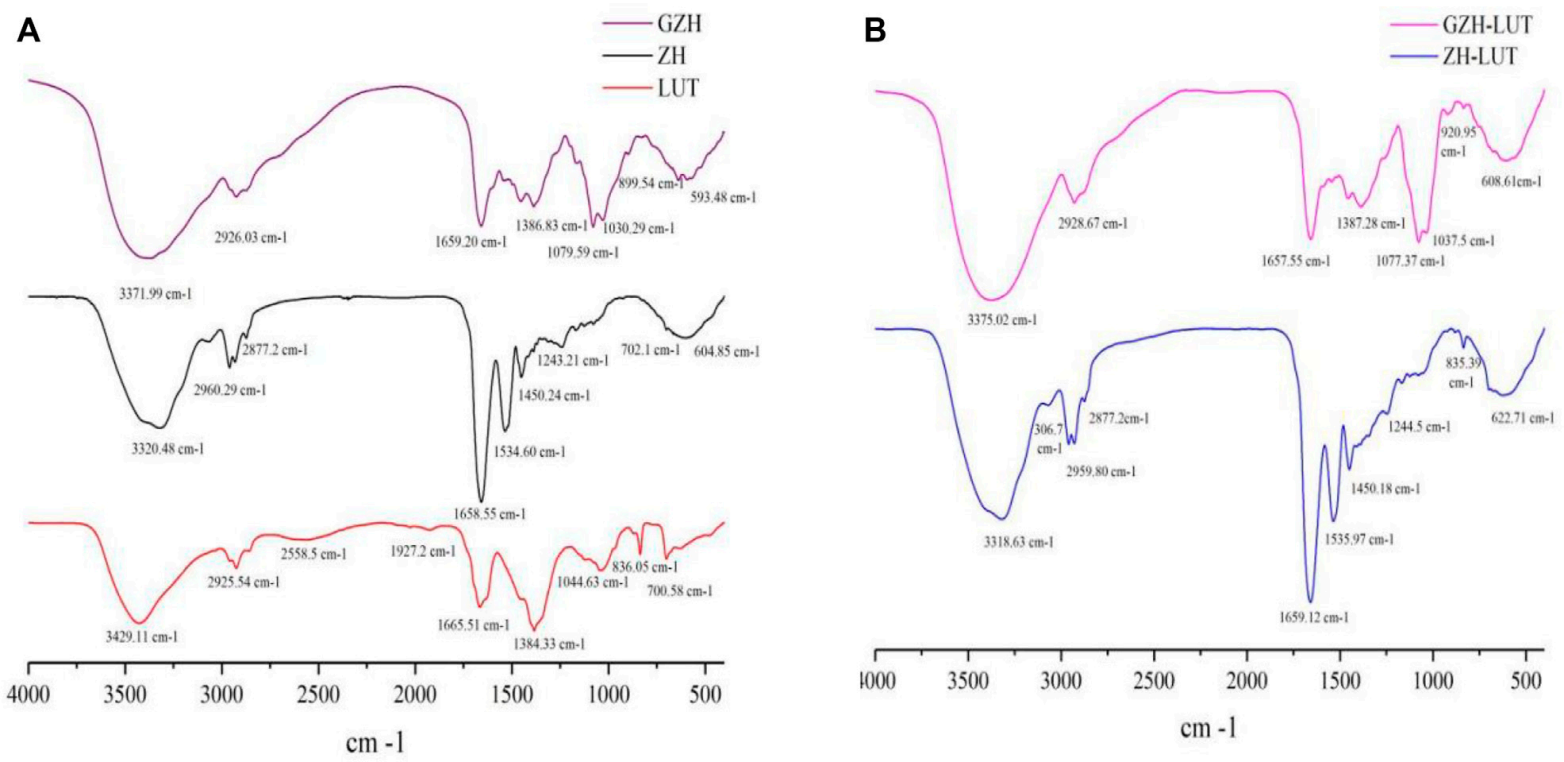
FTIR spectra of LUT, ZH and GZH carriers **(A)** and ZH-LUT and GZH-LUT nanoparticles **(B)**.

The surface chemistry of lutein, ZH carriers, GZH carriers, ZH-LUT nanoparticles and GZH-LUT nanoparticles was evaluated using FTIR spectroscopy (Figure 5). Lutein had a strong and wide absorption peak at 3,200–3,600 cm^−1^, which was caused by the stretching vibration of -OH (Toragall et al., 2020). In the spectra of the ZH-LUT nanoparticles and GZH-LUT nanoparticles, the peak shape and position of this band changed (Figures 5A,B), which indicates that hydrogen bonding occurred between the lutein, ZH and GZH carriers. In addition, lutein had typical C-H extension bands at 2,958 cm^−1^ and 2,912 cm^−1^, and the stretching vibration of C=C and the bending vibration of -CH_3_ caused the strong characteristic absorption peaks of lutein at 1,634.6 cm^−1^ and 1,384.33 cm^−1^ (Toragall et al., 2020), respectively. This behaviour was not observed in the spectra of the ZH and GZH carriers loaded with lutein, and there was no significant difference between ZH and GZH carriers before and after lutein was loaded. The characteristic absorption peak of lutein weakened or disappeared, indicating that there was no chemical reaction between lutein and the carrier after emulsification but only physical interaction, and the infrared spectrum results also confirmed that lutein was successfully loaded onto it (Shwetha et al., 2020).

Furthermore, in the FTIR spectra of ZH carriers and GZH carriers (Figure 5A), the stretching vibration absorption peaks of -OH in the range of 3,200–3,700 cm^−1^ and C-O in the range of 1,000–1,250 cm^−1^ became wider, indicating that -OH increased significantly after glycosylation and bound to sugar in a covalent bond (Wang X.-j. et al., 2020). When a sugar molecule was grafted to ZH, a new C-O bond was also introduced, and the infrared spectrum showed a new absorption peak at 1,000 cm^−1^ corresponding to the C-O bond (Zhang R. et al., 2022).

### 3.5 Solubility

**FIGURE 6:**
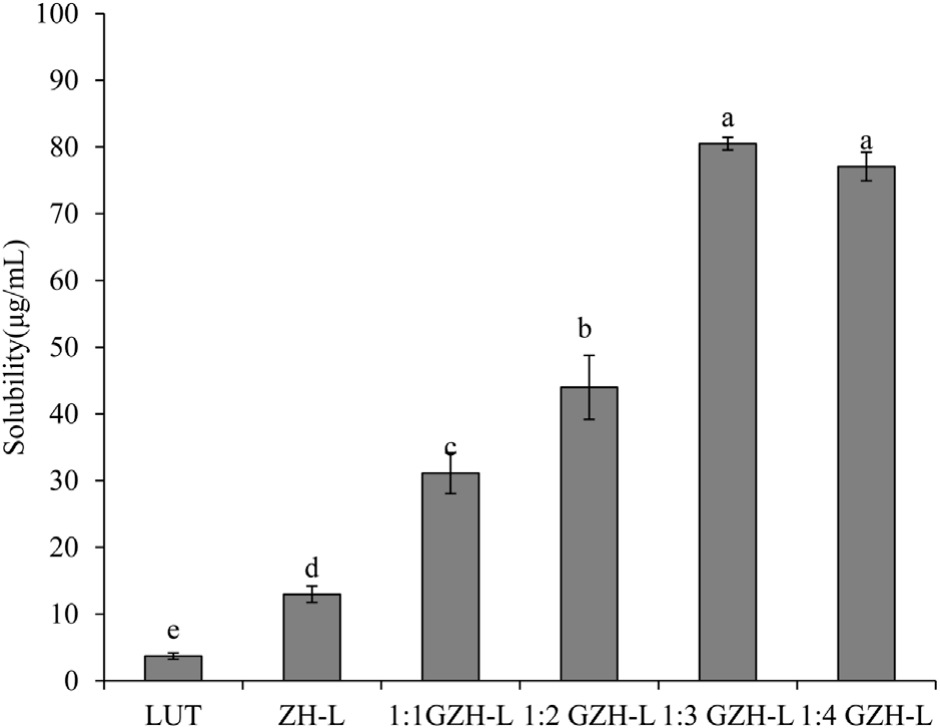
Solubility of LUT, ZH-LUT and GZH-LUT nanoparticles.

Lutein is a fat-soluble bioactive ingredient with low water solubility. After standing for a period of time, the free lutein precipitates and aggregates to different degrees, and the lutein aqueous solution appears turbid. When we used ZH carriers or GZH carriers with a certain solubility, the nanoparticle suspension formed after had a good dispersion effect the lutein was encapsulated, and almost no precipitation occurred with the extension of time. ZH carriers or GZH carriers improved the water solubility of lutein to varying degrees. At the same concentration of lutein, the solubility of unembedded lutein was the lowest, only 3.71 ± 0.47 μg/ml, and the solubility of lutein in ZH-LUT nanoparticles was approximately 3 times that of lutein. The solubility of GZH-LUT nanoparticles increased gradually with increasing glycosylation. When the mass ratio of protease hydrolysate to glucosamine was 1:3, the solubility of GZH-LUT nanoparticles was the highest, which was approximately 20 times that of unencapsulated lutein (Figure 6).

The difference in solubility was because in the preparation of nanoparticles by the reverse solvent method, lutein and ZH carriers or GZH carriers were combined through noncovalent interactions, such as hydrogen bonding, to form evenly distributed nanoparticles, thus inhibiting further aggregation and precipitation of lutein (Chen et al., 2020). After lutein was nanoencapsulated, the specific surface area of the formed ZH-LUT nanoparticles or GZH-LUT nanoparticles increased, which further promoted the dissolution and diffusion of lutein. It is also possible that after the nanoencapsulation of lutein, the molecular mobility of lutein was reduced, thereby improving the solubility or dispersibility of lutein (Liu et al., 2020b). The solubility of GZH-LUT nanoparticles was relatively high because in the process of glycosylation modification of ZH carriers, the number of hydrophilic carboxyl groups increased due to the covalent binding of glucose to the ZH carriers to improve the solubility of the GZH carriers, the internal encapsulation of lutein had a solubilizing effect granting the loaded lutein the highest solubility.

### 3.6 Stability to Environmental Stresses

#### 3.6.1 Heat Stability

**FIGURE 7:**
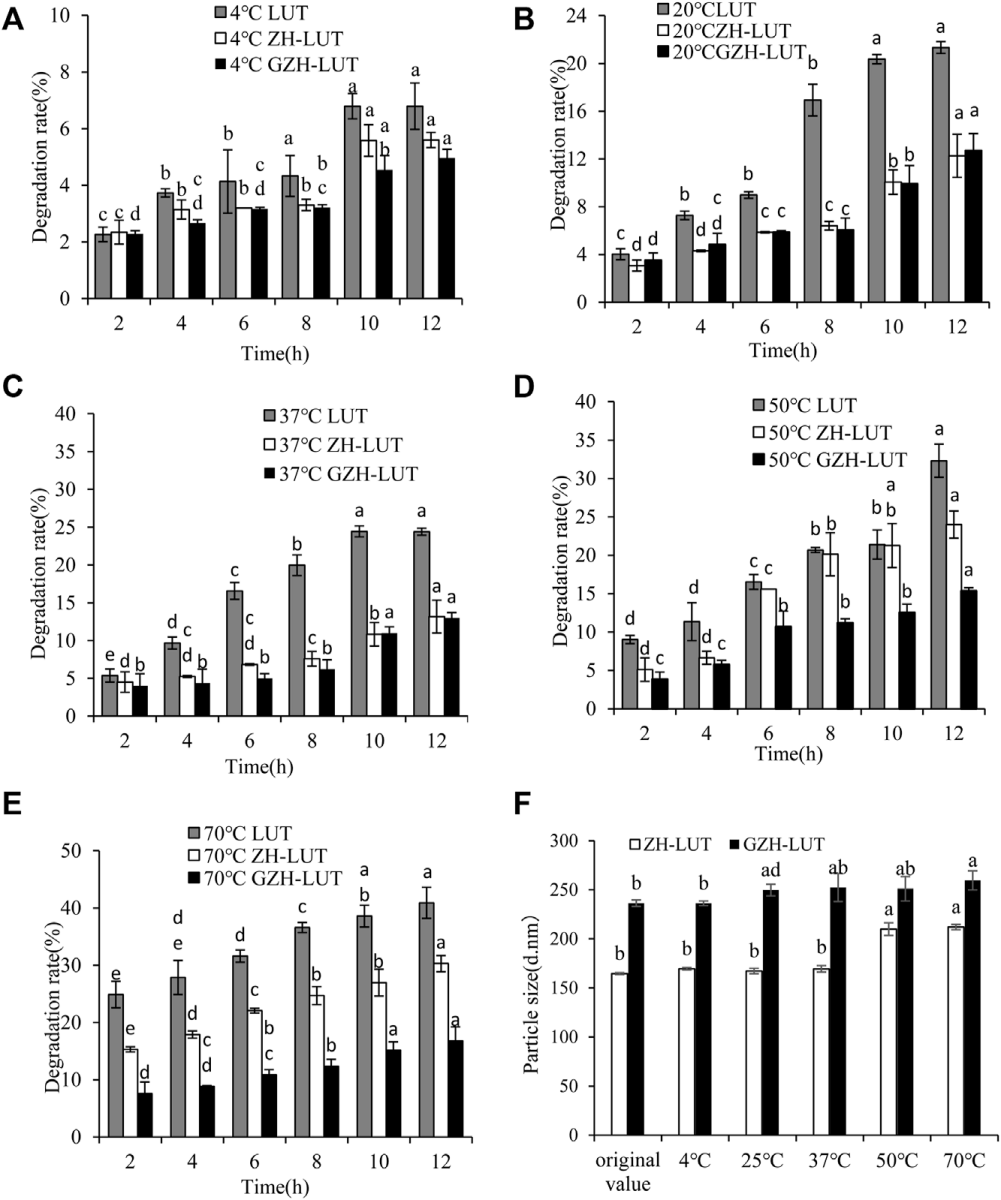
Effects of temperature on the heat stability of free lutein, ZH-LUT and GZH-LUT nanoparticles **(A−F)**.

Temperature has a great influence on different forms of lutein. At 4°C, the lutein and lutein nanoparticles were both stable. With increasing temperature, the degradation rate of the free lutein gradually accelerated, and the degradation rate of both lutein nanoparticles was relatively slow. This result shows that the sensitivity of lutein to temperature was reduced after encapsulation of lutein. However, when the temperature was increased to 70°C, the degradation rates of free lutein and that in the ZH-LUT nanoparticles gradually increased, reaching 40.9 ± 2.72% and 30.3 ± 1.41%, respectively. The degradation rates of lutein in the GZH-LUT nanoparticles were lower, only 16.8 ± 2.43%, the protective effect of the GZH carriers on lutein was better (Figure 7).

The measurement of DLS showed that the size of the ZH-LUT nanoparticles and GZH-LUT nanoparticles changed little at 4°C, 20 and 37°C, indicating that the nanoparticles were stable. However, when the temperature was above 50°C, varying degrees of agglomeration occurred between the nanoparticles. The size of the ZH-LUT nanoparticles increased, and the stability of nanoparticles decreased, while the particle size range of GZH-LUT nanoparticles always changed little. The reason may be that the nanocarriers after glycosylation modification generated thermally stable structures and increased the steric hindrance and net surface charge in the nanosystem. The protective effect of the GZH carriers on lutein was better (Fan et al., 2017; Swetledge et al., 2021; Tan et al., 2021).

#### 3.6.2 pH Stability

**FIGURE 8:**
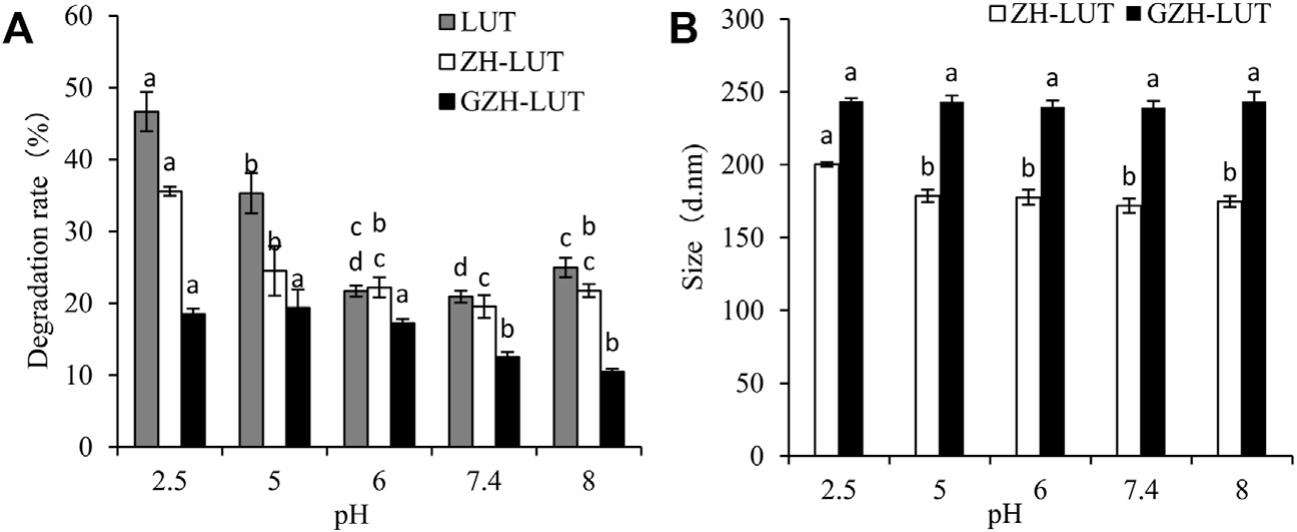
Effects of pH on the pH stability of free lutein, ZH-LUT and GZH-LUT nanoparticles **(A,B)**.

Under extremely acidic conditions, the rate of lutein degradation reached 46.65 ± 2.74%, indicating that the structure of lutein had a significant effect under extremely acidic conditions, and the stability of lutein was poor. The rate of lutein degradation in the ZH carriers was also higher, which was 35.58 ± 0.62%, and that of the lutein in the ZH-LUT nanoparticles changed from 171 ± 4.9 nm to 200.2 ± 1.51 nm, the particle size became larger, and the nanoparticles were relatively unstable (Figure 8).

At low pH, acid hydrolysis damaged the ZH carriers, resulting in the exposure of lutein, and after glycosylation modification, the resistance to strong acids was improved, and the degradation of encapsulated lutein was lower. Moreover, the GZH nanoparticles were very stable with almost no change in particle size, indicating that the GZH carriers could protect lutein from degradation (Ren et al., 2017; Razi et al., 2018; Xu G. et al., 2020).

#### 3.6.3 Light Stability

**FIGURE 9:**
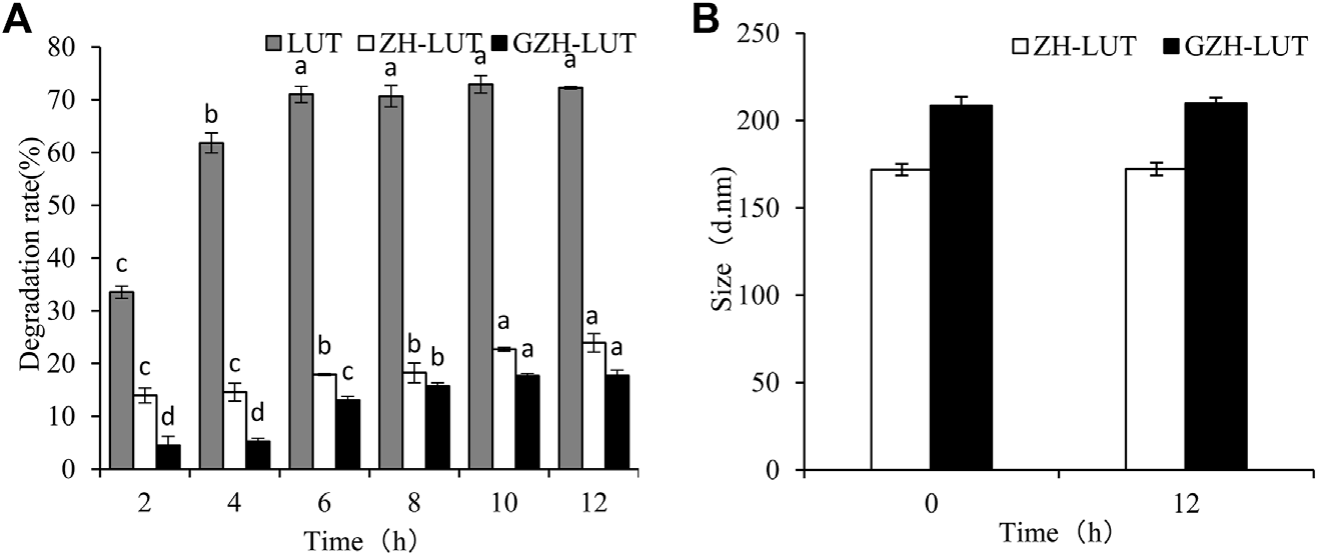
Effects of light radiation on the stability of free lutein, ZH-LUT and GZH-LUT nanoparticles **(A,B)**.

Lutein is very sensitive to light and protects the eye from retinal oxidative stress caused by intense exposure to UV light, but it is easily degraded under UV light. Figure 9 shows that lutein was easily degraded at different UV irradiation times, but the degradation rate of lutein was relatively slow after encapsulation. After 12 h of UV light irradiation, the rate of lutein degradation reached 72.26 ± 0.23%, and the lutein degradation rates of ZH-LUT nanoparticles and GZH-LUT nanoparticles were lower, only 23.91 ± 1.75% and 17.7 ± 1.04%, respectively (Figure 9). This result shows that the nanoencapsulation of lutein can significantly improve the photostability of lutein itself under the protection of the carrier because both the ZH carriers and the GZH carriers scattered and blocked the light, preventing the loss of lutein under UV irradiation (Li H. et al., 2020; De Boer et al., 2020). However, the size of the ZH-LUT nanoparticles and GZH-LUT nanoparticles did not change, indicating that light did not affect the size of the lutein nanoparticles.

### 3.7 Antioxidant Capacity

**FIGURE 10:**
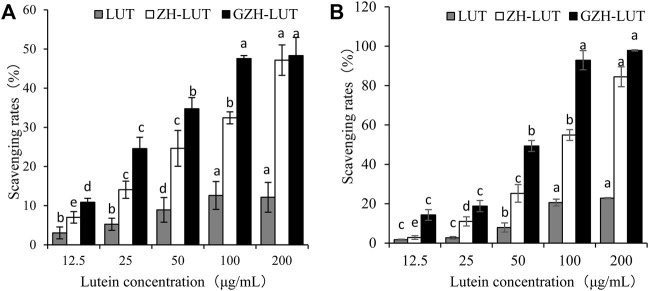
Rates of DPPH radical **(A)** and hydroxyl radical **(B)** scavenging by lutein, ZH-LUT nanoparticles and GZH-LUT nanoparticles.

The DPPH radical and hydroxyl radical scavenging abilities of LUT, ZH-LUT nanoparticles and GZH-LUT nanoparticles are shown in Figure 10. The DPPH radical and hydroxyl radical scavenging abilities were enhanced with increasing concentrations for all samples. At the same concentration, the antioxidant capacity of lutein was lower than that of the two lutein nanoparticles. When the concentration of lutein reached 200 μg/ml, the rate of DPPH clearance by the GZH-LUT nanoparticles reached 48.33 ± 4.6%, which was approximately 4 times that by free lutein. The results showed that lutein was combined with the carriers, and the antioxidant capacity was significantly improved (Figure 10). This result may have two explanations: when lutein was embedded in the ZH carriers and GZH carriers, the influence of the external environment on lutein was reduced, and the oxidant stability was improved, but free lutein was unstable and was lost or inactivated to a certain extent during the preparation process; therefore, the antioxidant activity of free lutein was low (Yi et al., 2016). In contrast, the ZH carriers and GZH carriers themselves had antioxidant capacity, and the overall antioxidant capacity was enhanced after they were combined with lutein to form nanoparticles (Carvalho et al., 2015; Rai et al., 2015; Ren et al., 2017).

The DPPH scavenging rate of GZH-LUT nanoparticles was higher than that of ZH-LUT nanoparticles, which may be due to the improved hydrogen supply capacity of the GZH-LUT nanoparticles after glycosylation modification and the grafting of sugar molecules on the surface of the ZH carriers, which improved the antioxidant capacity of the ZH carriers (Betbeder et al., 2015; Jiang et al., 2020; Liang et al., 2021). The hydroxyl radical scavenging rate of GZH-LUT nanoparticles was consistent with the measurement results of DPPH free radicals.

## 4 Conclusion

In conclusion, the present work demonstrated that the zein hydrolysate was glycosylated to prepare a nanodelivery carrier for lutein. Lutein-loaded zein hydrolysate nanoparticles (ZH-LUT) and glycosylated zein hydrolysate nanoparticles (GZH-LUT) were successfully prepared by a liquid–liquid dispersion method. Compared with ZH-LUT nanoparticles, GZH-LUT nanoparticles improved the encapsulation efficiency of lutein and showed better microstructure, regularity and dispersity. The GZH carriers effectively enhanced the oxidation stability and environmental stability of lutein. In future work, we will focus on the biological activity and cellular absorption mechanism of GZH-LUT nanoparticles.

## Data Availability

The original contributions presented in the study are included in the article/Supplementary Material, further inquiries can be directed to the corresponding author.
